# Hole-Transport Management Enables 23%-Efficient and Stable Inverted Perovskite Solar Cells with 84% Fill Factor

**DOI:** 10.1007/s40820-023-01088-4

**Published:** 2023-04-30

**Authors:** Liming Liu, Yajie Ma, Yousheng Wang, Qiaoyan Ma, Zixuan Wang, Zigan Yang, Meixiu Wan, Tahmineh Mahmoudi, Yoon-Bong Hahn, Yaohua Mai

**Affiliations:** 1 Institute of New Energy Technology, College of Information Science and Technology, Jinan University, Guangzhou, 510632 People’s Republic of China; 2 Guangdong Mellow Energy Co., Ltd., Guangzhou, 510630 People’s Republic of China; 3 Key Laboratory of New Semiconductors and Devices of Guangdong Higher Education Institutes, Jinan University, Guangzhou, 510632 People’s Republic of China; 4 School of Semiconductor and Chemical Engineering, Solar Energy Research Center, Jeonbuk National University, 567 Baekjedaero, Deokjin-gu, Jeonju-si, Jeollabuk-do 54896 Republic of Korea

**Keywords:** Inverted NiO_*x*_-based perovskite solar cells, Hole-transport management, Interface-induced defect passivation, High performance and stability

## Abstract

NiO_*x*_-based inverted perovskite solar cells (PSCs) have presented great potential toward low-cost, highly efficient and stable next-generation photovoltaics. However, the presence of energy-level mismatch and contact-interface defects between hole-selective contacts (HSCs) and perovskite-active layer (PAL) still limits device efficiency improvement. Here, we report a graded configuration based on both interface-cascaded structures and p-type molecule-doped composites with two-/three-dimensional formamidinium-based triple-halide perovskites. We find that the interface defects-induced non-radiative recombination presented at HSCs/PAL interfaces is remarkably suppressed because of efficient hole extraction and transport. Moreover, a strong chemical interaction, halogen bonding and coordination bonding are found in the molecule-doped perovskite composites, which significantly suppress the formation of halide vacancy and parasitic metallic lead. As a result, NiO_*x*_-based inverted PSCs present a power-conversion-efficiency over 23% with a high fill factor of 0.84 and open-circuit voltage of 1.162 V, which are comparable to the best reported around 1.56-electron volt bandgap perovskites. Furthermore, devices with encapsulation present high operational stability over 1,200 h during T_90_ lifetime measurement (the time as a function of PCE decreases to 90% of its initial value) under 1-sun illumination in ambient-air conditions.

**Supplementary Information:**

The online version contains supplementary material available at 10.1007/s40820-023-01088-4.

## Highlights

- A graded inverted solar cell configuration is developed by hole-transport management aiming to suppress interface defects-induced non-radiative recombination for efficient hole transport.
- NiO_*x*_-based inverted PSCs present a power-conversion-efficiency over 23% with a high fill factor of 0.84 and open-circuit voltage of 1.162 volts, one of the best performances reported so far for 1.56-electron volt bandgap formamidinium-based triple-halide perovskites.
- Devices show high operational stability over 1,200 h during T_90_ lifetime measurement under 1-sun illumination in ambient-air conditions.

**Supplementary Information:**

The online version contains supplementary material available at 10.1007/s40820-023-01088-4.

## Graphical abstract

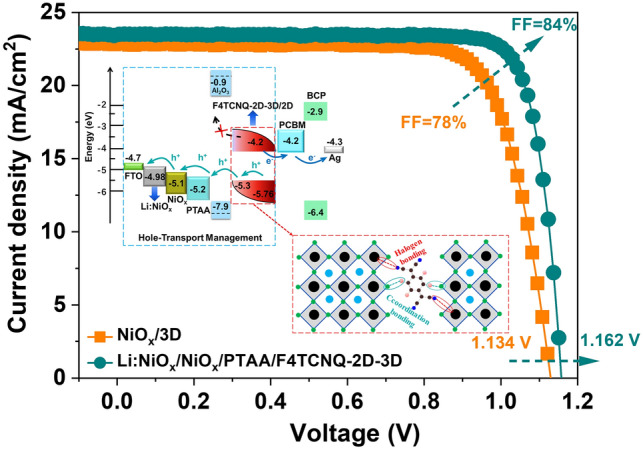

## Introduction

Although metal halide perovskites (MHPs) with formula of ABX_3_ (A = monovalent cation, B = metallic divalent cation and X = halide anion) experienced from emerging to shining star materials within just several decades, they have been considered as one of the most potential next semiconductor materials for the development of solution-processed, high-performance and low-cost optoelectronic devices [1–6]. The advent of solid-state perovskite solar cells (PSCs) since 2012 [7, 8], we have witnessed their great success in the third-generation/emerging photovoltaics [9–13]. Undoubtedly, PSCs have a great opportunity to become a game-changer in the photovoltaics market. Due to their marvellous optical and electronic properties [14–17], single-junction PSCs, all-perovskite tandem cells and perovskite-silicon tandem cells, etc., have demonstrated an incredibly rapid ascent in PCE, reaching a record efficiency of 25.73% [18], 28% [19] and 32.5% [20], respectively. Despite much effort, unluckily, device operational stability has been main bottleneck for further development. For example, external encapsulation strategy as a necessary craft process not only can prevent the infiltration of moisture and oxygen, but also enable lead sequestration, thus enhancing the environmental stability of perovskites [21–23]. Nevertheless, PSCs treated under continuous light illumination still show poor operational stability, though devices were dealt with external encapsulation. This is because light or heat can induce the escape of organic compounds, halide ion migration or segregation from perovskites, leading to the formation of A-site and X-site vacancy defects [24–27]. Besides, migratory halide ions also can react with metal electrodes, generating metal halide compounds (such as AgI_2_^−^/AuI_2_^−^ and AgCl_2_^−^/AuCl_2_^−^) at heterointerfaces in a solar cell [28–31]. Quite remarkably, robust interfaces and manipulation of the perovskite active layer in the stacked PSC configurations are highly desirable [32–34].

As is widely accepted, low-cost, thermal and chemical stable inorganic nickel oxide (NiO_*x*_) has been considered as one of most efficient hole transport materials to develop operational stable PSCs [30, 35–41]. Furthermore, NiO_*x*_ based inverted PSCs also present highly compatibility for development of flexible or tandem solar cells [42–44]. However, most of reported NiO_*x*_ based inverted PSCs still show relative low efficiency (~ 22%) due to the presence of contact-interface defects at transparent-conductive-oxide (TCO)/NiO_*x*_ and NiO_*x*_/perovskite heterointerfaces. Such imperfect heterointerface contacts, including perovskite buried interface and NiO_*x*_ surface defects, mismatched energy band levels, and undesirable interfacial chemical reactions, etc., can cause carrier accumulation and ineffective charge transport at stacked cell interfaces, resulted in non-radiative recombination, contact interface collapse and mechanical delamination issues. Thus, the heterointerface contacts not only determine charge injection and extraction dynamics, but also affect device operational stability. Especially, the heterointerface defects severely limit the device open-circuit voltage (*V*_oc_) and fill factor (FF) [45, 46]. Therefore, understanding heterointerface properties and design of energy-level structures are of paramount importance for highly efficient and stable NiO_*x*_-based inverted perovskite photovoltaics.

Herein, we design a graded p-type side contact interfaces based on both efficient interface-cascaded structures and p-type molecule-doped composites with two-/three-dimensional (2D/3D) perovskites aiming to reduce energy-level mismatch, contact-interface defects and energetic barriers across the heterointerface. UV–visible and ultraviolet photoelectron spectrum measurements are used to confirm an efficient energy level alignment of the p-type side contact interfaces (i.e., FTO/Li:NiO_*x*_/NiO_*x*_/PTAA/Al_2_O_3_/ composite-based F4TCNQ-3D-2D/2D perovskites), facilitating charge extraction and hole transportation, and reducing nonradiative recombination from the perovskite layer to hole transport layers and to TCO layer. By X-ray photoelectron spectroscopy (XPS) and Fourier-transform infrared measurements combined with theoretical calculation, it is confirmed the presence of halogen bonding F…I and coordination bonding N…Pb between perovskites and F4TCNQ molecules, which effectively mitigate the formation of halide vacancy and parasitic metallic Pb^0^. Compared to the NiO_*x*_ HTL based control cells, the champion devices with efficient p-type side contact interfaces showed dramatic enhancement of photovoltaic parameters and operational stability.

## Experimental Section

### Materials

Lithium carbonate (Li_2_CO_3_, ≥ 99.99%) was purchased from MACKLIN, Shanghai. Nickel (II) Acetate Tetrahydrate (Ni(OCOCH_3_)_2_·4H_2_O (99.9%), mesoporous Al_2_O_3_ dispersion (30 nm, 20 wt% in isopropanol), dimethyl sulfoxide (DMSO, > 99.9%), N, N-dimethylformamide (DMF, 99.8%) and chlorobenzene (CB, 99.8%) were purchased from Sigma-Aldrich. Lead bromide (PbBr_2_, 99.99%), Lead iodide (PbI_2_, 99.99%), Cesium iodide (CsI, 99.99%), Poly[bis(4-phenyl) (2,4,6-trimethylphenyl) amine (PTAA), 2,3,5,6-tetrafluoro-7,7,8,8-tetracyanoquinodimethane (F4TCNQ, > 99%), oleylammonium iodide (OAmI, > 99.5%), Formamidinium iodide (FAI, ≥ 99.5%), methylammonium bromide (MABr, > 99.5%) and phenethylammonium (PEAI, > 99.5%) were purchased from Xi’an p-OLED Corp. Isopropanol (IPA, ≥ 99.9%), ethanol (99.5%) and ethyl acetate (EA, ≥ 99%) were purchased from Aladdin. BCP and PCBM were purchased from Lumtec. Ethanolamine (99%) was purchased from Alfa Aesar.

### Preparation of Perovskite Precursor

The perovskite precursor solution was prepared by dissolving 0.218 g FAI, 0.014 g MABr, 0.045 g PbBr_2_, 0.024 g MACl, 0.643 g PbI_2_ and 0.002 g OAmI into a mixed solvent of 0.8 mL DMF and 0.2 mL DMSO. Then, different contents of F4TCNQ added into above solution and followed by further heat treatment at 40 °C for 10 min.

### Devices Fabrication

FTO substrates were cleaned with sonication in detergent, diluted water, IPA and ethanol each for 20 min. After dried with N_2_ flow, the FTO substrates were further heated at 200 °C for 5 min to remove all residual organic materials and then followed by oxygen plasma treatment for 10 min. The Li_2_CO_3_ solution was prepared by dissolving 3 mg Li_2_CO_3_ into the mixture solvents with ethanol and deionized water. The Li doped NiO_*x*_ precursor solution was prepared by adding different contents of Li_2_CO_3_ solution to NiO_*x*_ precursor solution (The prepared method of NiO_*x*_ precursor solution can be found in previous work [30, 40]. The Li doped NiO_*x*_ precursor solution was spin coated on as-cleaned FTO substrates at 4,000 rpm for 30 s to form FTO/Li:NiO_*x*_ film, and then heated at 200 °C for 40 min. Next, NiO_*x*_ precursor solution was further coated on the FTO/Li:NiO_*x*_ film, and annealed at 400 °C for 40 min. After oxygen plasma treatment of FTO/Li:NiO_*x*_/NiO_*x*_ films for 2 min, the PTAA solution and mesoporous Al_2_O_3_ (mp-Al_2_O_3_) solution were spin coated successively on the FTO/Li:NiO_*x*_/NiO_*x*_ substrates; then, As-prepared perovskite precursor solution was spin coated on the FTO/Li:NiO_*x*_/NiO_*x*_/PTAA/mp-Al_2_O_3_ substrate and antisolvent treatment by EA, followed by deposition of PEAI solution. The PCBM and BCP were coated successively on the above films. Finally, Ag electrode was deposited by a shadow mask in a thermal evaporator (QHV-R53) under high vacuum.

### Characterization

The surface morphology and cross-sectional view of perovskite films were examined by scanning electron microscopy (SEM, FEI Apreo LoVac). The crystal structure, absorption properties, elemental compositions and chemical and electron states of perovskite films were analyzed with X-ray diffractometer (XRD, Bruker D8 Advance), Grazing-Incidence Wide-Angle X-ray Scattering (GIWAXS), ultraviolet–visible (UV–vis, DektakXT), X-ray photoelectron spectroscopy (XPS, AXIS ULTRA DLD, aluminum Kα X-ray radiation) and Fourier transform infrared (FTIR, Thermo) transmittance measurements, respectively. The focused-ion-beam (FIB)-butted 2D-3D/2D perovskite films are examined by high resolution transmission electron microscopy (HR-TEM). The photoluminescence (PL) and time-resolved photoluminescence (TRPL) spectra of perovskite films were examined by using an Edinburgh Instruments FL1000 fluorescence spectrometer with 532 nm excitation source.

The performance of all devices without encapsulation were carried out under ambient-air conditions. The current density–voltage (*J-V*) curves and photovoltaic parameters of PSCs were measured by a Newport solar simulator (Keithley series 2400, ORIEL-SOI3A) with a source meter at 100 mW cm^−2^ under AM 1.5 illumination which was calibrated by a silicon reference cell. The solar cell performances were examined with 0.09 cm^2^ mask/aperture. The external quantum efficiency (EQE) spectrum was measured by using a QE-R instrument from Enlitech, calibrated by a silicon reference cell. The dynamic I-V curves of hole-only and electron-devices were examined by using a semiconductor analyzer (ZAHNR CIMPS-2) at voltage range from 0 to 2 V, current range from − 2 to 2 A, and scan rate at 100 mV s^−1^ under room temperature. The long-term stability of cells with encapsulation was tested under continuous one-Sun illumination in ambient-air conditions.

## Results and Discussion

### Characterization of FTO/Li:NiO_***x***_/NiO_***x***_/PTAA Hole-Contact Structure

**Fig. 1:**
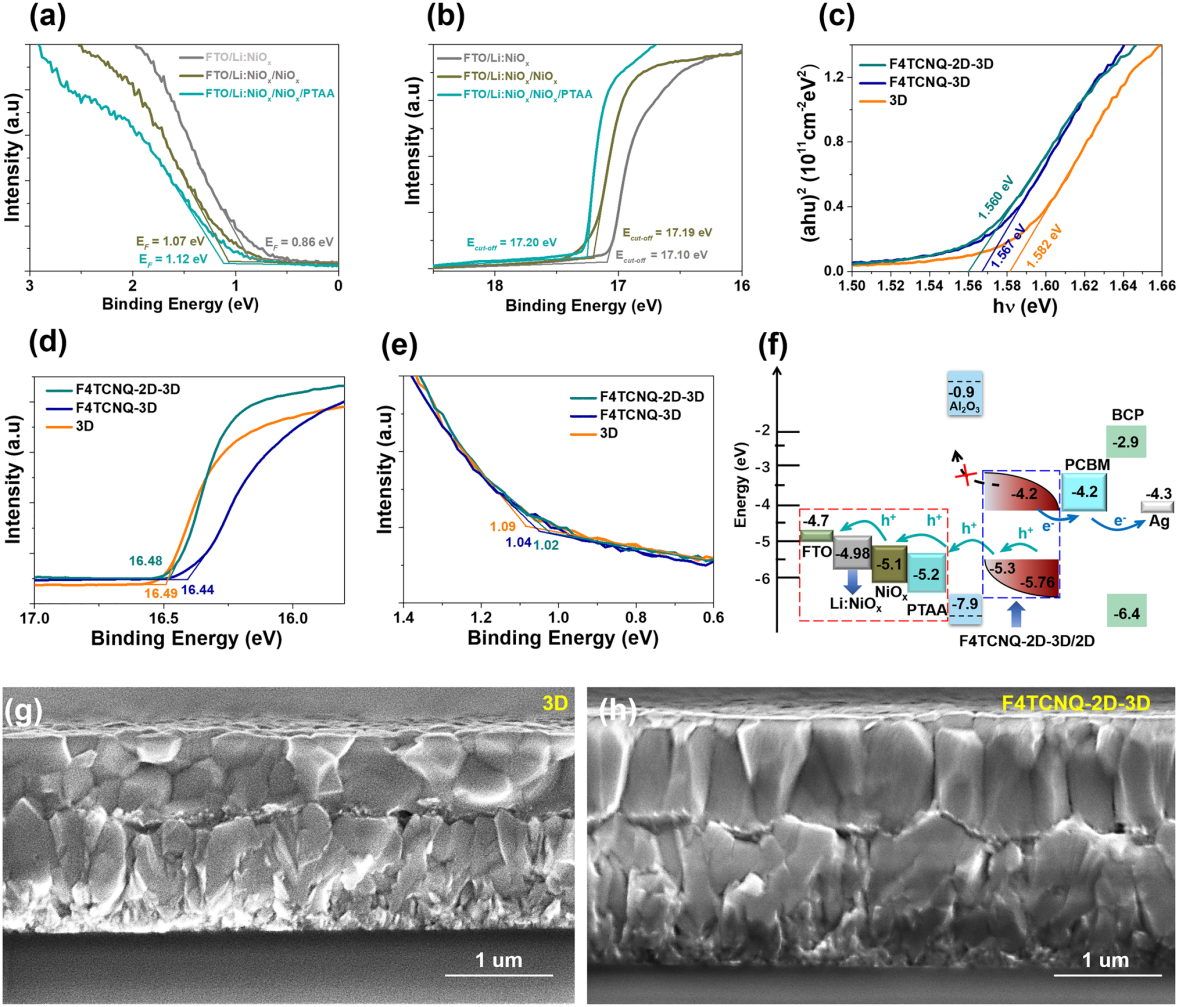
**a–b** UPS of the Fermi level (E_*F*_) energy and corresponding cutoff (E_*cut-off*_) regions for FTO/Li:NiO_*x*_, FTO/Li:NiO_*x*_/NiO_*x*_ and FTO/Li:NiO_*x*_/NiO_*x*_/PTAA. **c** Tauc plots and **d–e** UPS of three perovskite films: 3D (orange), F4TCNQ-3D composite (royal) and F4TCNQ-2D-3D composite (dark cyan). **f** Device energy band diagram by hole-transport management strategy. **g–h** Cross-sectional SEM images of pristine 3D perovskite and F4TCNQ-2D-3D composite films

To maximumly reduce energy-level mismatch induced hole-transport deficiency from hole transport layer (HTL) to TCO, an interface-cascaded structure with p-type contacts is designed, as shown in Fig. 1f (red dotted box). The optical and electronic properties of three films, i.e., FTO/lithium doped NiO_*x*_ (Li:NiO_*x*_), FTO/Li:NiO_*x*_/NiO_*x*_ and FTO/Li:NiO_*x*_/NiO_*x*_/PTAA (PTAA: Poly[bis(4-phenyl) (2,4,6-trimethylphenyl) amine), can be determined by UV–visible (UV–Vis) and ultraviolet photoelectron spectrum (UPS) (Figs. S1 and 1a-b). Note that three films show almost same transmittance in the wavelength range of 400–850 nm (Fig. S1), indicating that such a p-type contact structure does not reduce light harvesting by the perovskite active layer (PAL) (Fig. S2a). To confirm efficient energy level diagram in the FTO/Li:NiO_*x*_/NiO_*x*_/PTAA film, the valence band (VB) of three films are calculated to be − 4.98, − 5.1 and − 5.2 eV by the equation of 21.22 eV (excitation energy) – (E_cut-off_–E_*F*_) [40, 47]. Such a cascaded energy-level diagram facilitates hole extraction and transport from the perovskite to HTL and FTO layer. In this work, we further use insulator mesoporous alumina (mp-Al_2_O_3_) as a scaffold which not only can improve the contact interface at PTAA/perovskite but also facilitate charge extraction and transport by effective electron blocking represented by dashed black arrows (Fig. 1f). Therefore, the FTO/Li:NiO_*x*_/NiO_*x*_/PTAA/mp-Al_2_O_3_ structure does not lower hole-transport efficiency, adversely it substantially reduces non-radiative recombination, resulting in efficient charge carriers separation and transport in the perovskite layer and hole-contact interfaces.

### Structural Characterization and Optoelectronic Properties of Composite-Based F4TCNQ-2D-3D/2D Film

**Fig. 2:**
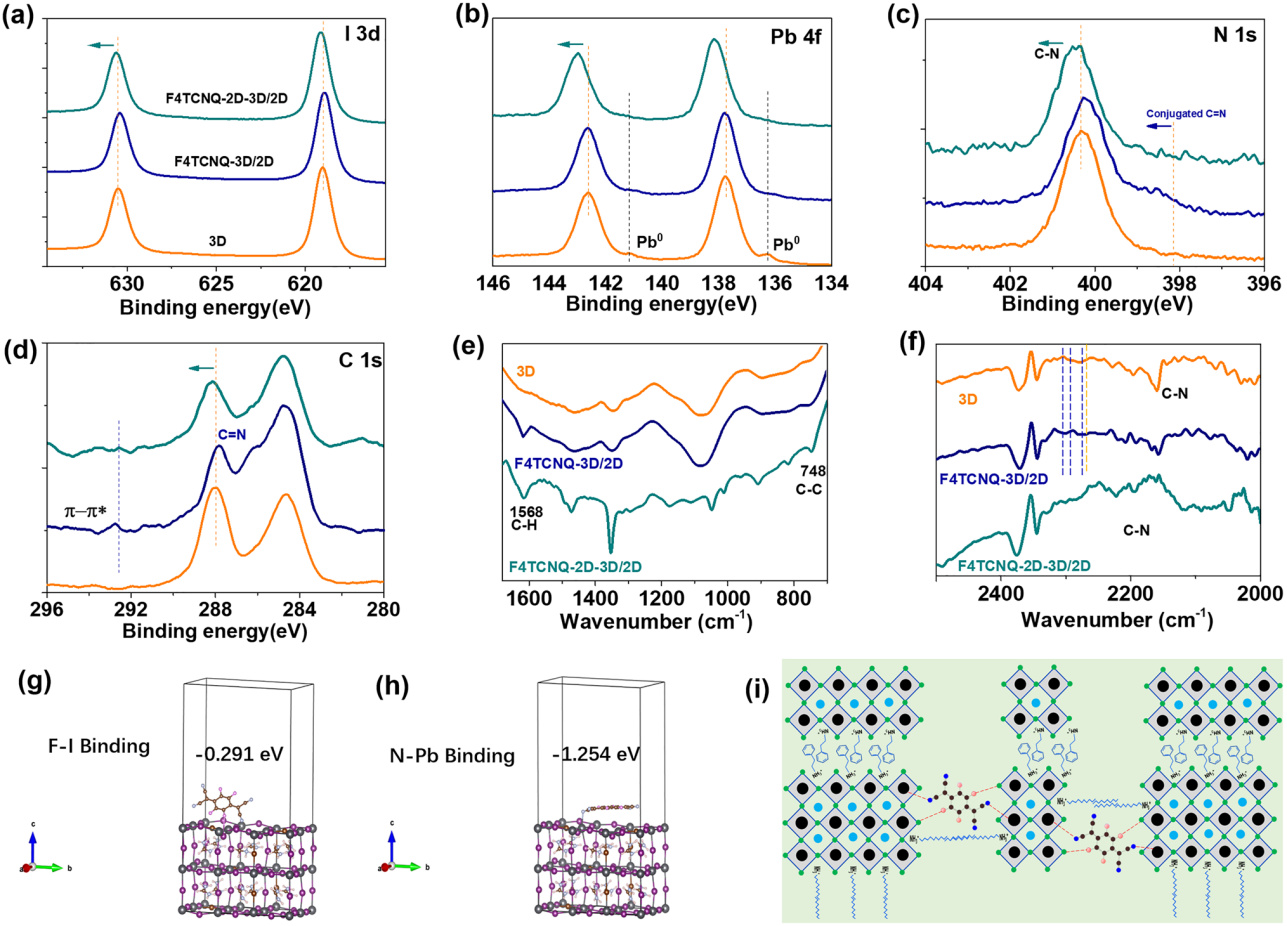
**a–d** XPS spectra of I 3*d*, Pb 4*f*, N 1*s* and C 1*s*, **e–f** FTIR spectra at 700–1680 cm^−1^ and 2000–2500 cm^−1^ for 3D (orange), F4TCNQ-3D/2D composite (royal) and F4TCNQ-2D-3D/2D composite (dark cyan), respectively. **g–h** Binding energies for F…I and N…Pb coordination bonds by DFT calculation. **i** Schematic illustration of 2D/3D perovskite formation, and F…I and N…Pb chemical bonding between F4TCNQ and perovskites

In order to further enhance hole extraction and transport from the PAL to HTL, we developed a composite-based PAL incorporated with 2D-3D formamidinium-based triple-halide perovskites and p-type organic molecule 2,3,5,6-tetrafluoro-7,7,8,8-tetracyanoquinodimethane (F4TCNQ). The p-type F4TCNQ presents several important roles in the composite PAL: (1) to facilitate hole extraction due to high electron affinity [42]; (2) to protect perovskites from moisture due to the intrinsic hydrophobic character of F4TCNQ [48]; (3) to suppress the defects of perovskites by coordinating undercoordinated Pb^2+^ clusters by a pair of nonbonding electrons (N atom) (Fig. 2) [49, 50]; (4) to build a graded energy structure due to closer valence bands between perovskite and F4TCNQ, as shown in Fig. 1f (blue dotted box). It is worth mentioning that we selected long-chain alkylamine ligands, oleylammonium (OAm), to build 2D-3D bulk heterostructures (2D: (OAm)_2_PbI_4_) that were systematically studied in our previous work [40]. The 2D-3D bulk heterostructures showed better optical and electrical properties than the pristine 3D perovskites, thus we mainly compare the properties of pristine 3D, F4TCNQ-3D composite and F4TCNQ-2D-3D composite perovskites in this work. To confirm the role of energy-level arrangement by F4TCNQ in the PAL, UV–Vis spectra and UPS of 3D (orange), F4TCNQ-3D composite (royal) and F4TCNQ-2D-3D composite (dark cyan) films were measured, as shown in Figs. S2a-b and 1c-e. Compared to pristine 3D (FA_0.93_MA_0.07_Pb(I_0.92_Br_0.08_)_3-x_Cl_x_) perovskite films, perovskite composite-based films incorporated with F4TCNQ show a narrow band gap of 1.56 eV (Fig. 1c) and slightly shifted band-edge (Fig. S2a-b). Figure 1d-e clearly show the cutoff (*E*_cut-off_) and Fermi level (*E*_*F*_) energy regions. The VB of 3D, F4TCNQ-3D and F4TCNQ-2D-3D are estimated to be − 5.85, − 5.82 and − 5.76 eV. According to the band gap obtained from Tauc plots, the conduction band (CB) of the F4TCNQ-2D-3D perovskite-based composite films can be calculated to − 4.2 eV, indicating free electron transport barrier at the perovskite/PCBM interface. According to the above analysis, device energy band diagram is carefully illustrated in Fig. 1f.

To examine roles of F4TCNQ and 2D perovskites on the performance of 3D perovskites, we further investigate the optical and electronic properties of perovskite-based composites, i.e., F4TCNQ-2D-3D film. Compared to pristine 3D perovskite films, the perovskite composite-based F4TCNQ-3D and F4TCNQ-2D-3D films show strong XRD peaks and low values of Full width at half maximum (FWHM) (Fig. S3). In addition, both main peaks at (110) and (200) slightly shifted towards higher angles for the composite-based F4TCNQ-3D and F4TCNQ-2D-3D films (Fig. S3b-c), indicating a strong interaction between F4TCNQ and perovskite molecules (Fig. 2). We have found that OAm ligands can facilitate the growth of perovskite crystals in our previous work [40]. Similarly, the composite-based perovskite films with F4TCNQ also present highly crystalline and large-size grains (Fig. S4). Compared to the pristine perovskite film, the composite-based F4TCNQ-2D-3D film presents highly oriented and vertically aligned crystal grains (Fig. 1g–h), which guarantees efficient charge transport in the vertical direction [51]. Besides, the relative large molecular size of F4TCNQ cannot enter into the lattice of perovskites [52], thus F4TCNQ additives are mostly located at the GBs and/or interfaces in the 2D-3D bulk perovskites, which can significantly passivate GBs and interface defects.

**Fig. 3:**
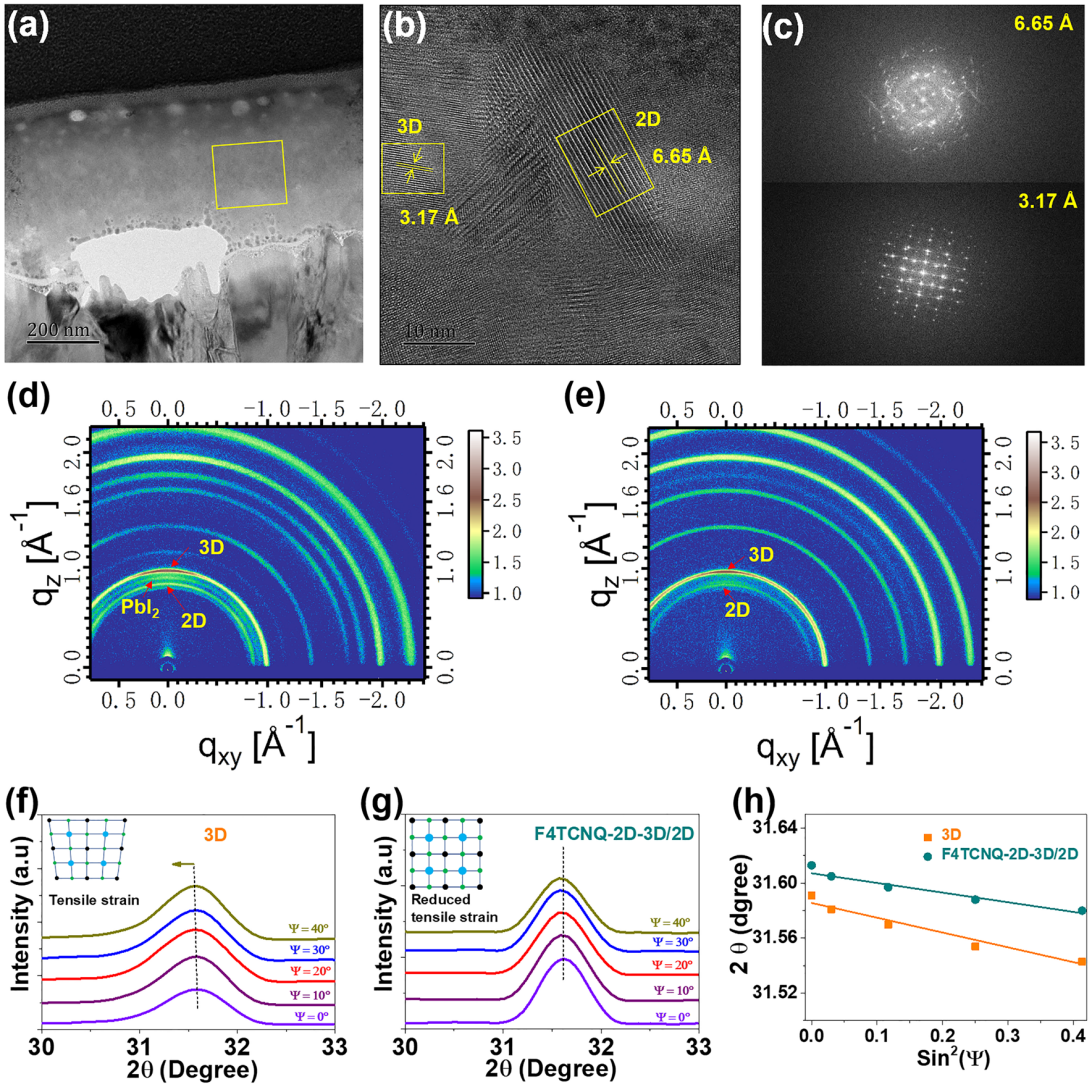
**a–c** FIB-cutted cross-sectional images for 2D-3D bulk perovskites, selected region of HR-TEM and corresponding inverse FFT images for 3D and 2D crystals, respectively. **d–e** GIWAXS images of 3D/2D heterointerface and 2D-3D-F4TCNQ composite films. **f–g** GIXRD spectra at different tilt angles for 2θ at 31.58° and **h** linear fit of residual strain as a function of sin^2^ψ for pristine 3D perovskite and 2D-3D-F4TCNQ composite films

To build 3D/2D perovskite heterointerfaces, the pristine 3D perovskite surface was treated by phenethylammonium (PEA) ligands, which is confirmed by focused-ion-beam (FIB)-assisted HR-TEM (Fig. S5) and grazing incidence wide-angle X-ray scattering (GIWAXS) measurements (Fig. 3d). The formation of 2D-3D/2D perovskite heterostructures can be clearly observed in HR-TEM images (Fig. 3a–c) and GIWAXS image (Fig. 3e). In the HR-TEM, two distinct lattice spaces of 3.21 and 6.65 Å can be observed in the composite-based perovskite bulk area, corresponding to 3D and 2D perovskites, respectively. Photoluminescence (PL) spectra performance further verify the formation of 2D perovskite due to the presence of extra weak peak at approximately 650 nm wavelength (Fig. S6a-b). Besides, the composite-based F4TCNQ-2D-3D film demonstrates a strong PL peak, i.e., 6 times higher than pristine perovskite (orange) films, indicating the formation of high-quality and homogenous perovskite crystals. Such results also are agreement with SEM performance (Figs. S4 and 1g-h). A slight red shift (785.8 → 795.6 nm) of the emission peak for composite-based F4TCNQ-2D-3D film is observed, which is ascribed to the reduced band gap (Fig. 1c). The strongest PL peak in the composite-based F4TCNQ-2D-3D film also suggests the suppression of the non-radiative recombination of charge carriers. Furthermore, the carrier life time is also determined by time-resolved PL (TRPL) according to exponential decay function [34, 40]:

1 $$F\left(t\right)={y}_{o}+{A}_{1}\mathrm{exp}(-t/{\tau }_{1})+{A}_{2}\mathrm{exp}(-t/{\tau }_{2})$$

where ${\tau }_{1}$ and ${\tau }_{2}$ are fast and slow decay time, ${A}_{1}$ and ${A}_{2}$ are amplitude fraction, respectively. According to fitting curves, average carrier life time (${\tau }_{\mathrm{ave}}$) of the pristine 3D, composite-based 3D-F4TCNQ and F4TCNQ-2D-3D films is calculated to 765, 1,028 and 1,144 ns (Fig. S6c), respectively. Such results indicate an efficient suppression of non-radiative recombination in composite-based perovskite films.

Then, we investigated the residual stress of pristine 3D perovskite (orange) and composite-based F4TCNQ-2D-3D films (dark cyan) by grazing-incidence XRD (GIXRD) spectra at different tilt angles for 2θ at 31.58° and 14.07° (Figs. S7 and in 3f–g). It can be observed that both crystallographic planes (i.e., 31.58° and 14.07°) shift towards lower 2θ positions, indicating the presence of lattice distortion and tensile stress within the pristine 3D perovskite film. However, the composite-based F4TCNQ-2D-3D film demonstrates almost same 2*θ* position for the two crystallographic planes when ψ angles change from 0° to 40°, as shown in Fig. 3f–g. Furthermore, linear fit curves for 2θ position as a function of sin^2^ψ in the composite-based F4TCNQ-2D-3D films have slower slopes than that of the pristine 3D perovskite films (Fig. 3h), indicating efficient suppression of the residual stress and lattice distortion. The presence of OAm^+^ ligands not only adjust the growth of 3D perovskite crystals, but also mitigate lattice distortion and residual stress due to the formation of 2D-3D heterostrutures [40, 47, 53].

### Chemical and Electronic States of Composite-Based F4TCNQ-2D-3D/2D Perovskite Film

As we mentioned earlier, perovskite film defects can be suppressed due to the presence of probable strong chemical interactions between F4TCNQ and perovskite molecules. To confirm the hypothesis, pristine 3D, F4TCNQ-3D and F4TCNQ-2D-3D perovskite films were carefully examined by X-ray photoelectron spectroscopy (XPS) and Fourier transform infrared (FTIR) spectra as illustrated in Fig. 2a–f. Compared to pristine 3D perovskite film, the core levels of all components in composite-based perovskite films incorporated F4TCNQ (i.e., I 3*d*, Pb 4*f*, N 1*s*, C 1*s*, Cl 2*p*, Br 3*d*, and F 1*s*) showed a substantial shift toward higher binding energy (Figs. 2a–d and S8). The shifted peak of core level of I 3*d* is attributed to a supramolecular interaction with halide ions (I…F) through halogen bonding [54–56], which can effectively matigate the formation of halide vacancy. No any metallic Pb^0^ peak can be found in the F4TCNQ-based perovskite composite films (Fig. 2b, royal and dark cyan curves), indicating efficient defect passivation in 3D perovskites by F4TCNQ and 2D perovskites. It is also observed that conjugated C=N bonds from N 1*s* and C 1*s* shifted towards higher binding energy, which probably contributes to the coordination bonding between undercoordinated Pb^2+^ clusters and N atom from F4TCNQ [49, 50]. The calculated binding energies by density functional theory (DFT) also further confirmed the presence of F…I and N…Pb coordination bonds (Fig. 2g–h). From C 1*s* XPS spectra (Fig. 2d), one shoulder peak approximately at 292.79 eV ascribed to π-π* satellite was observed, indicating the formation of 2D perovskites, and interface termination by PEA and OAm ligands in the functionalized crystals [40, 57]. In FTIR spectra, two new peaks assigned to aromatic C-H and aromatic C = C vibrational modes at 748 and 1568 cm^−1^ were observed (Fig. 2e), which further confirm the formation of 2D perovskites. Compared to the pristine 3D film, C-N stretching vibrations at 2159.24 cm^−1^ in the composite-based perovskite films slightly shifted toward a lower wavenumber (Fig. 2f). In addition, new peaks ascribed to C-N stretching vibrations at 2168.82, 2191.20 and 2208.41 cm^−1^ can be observed in the composite-based perovskite films (Fig. 2f). According to the XPS and FTIR spectra and binding energy calculations, we deduced the mechanism of chemical interaction and formation of 2D-3D/2D perovskites as shown in Fig. 2i.

### Solar Cell Photovoltaic Performance and Operational Stability

**Fig. 4:**
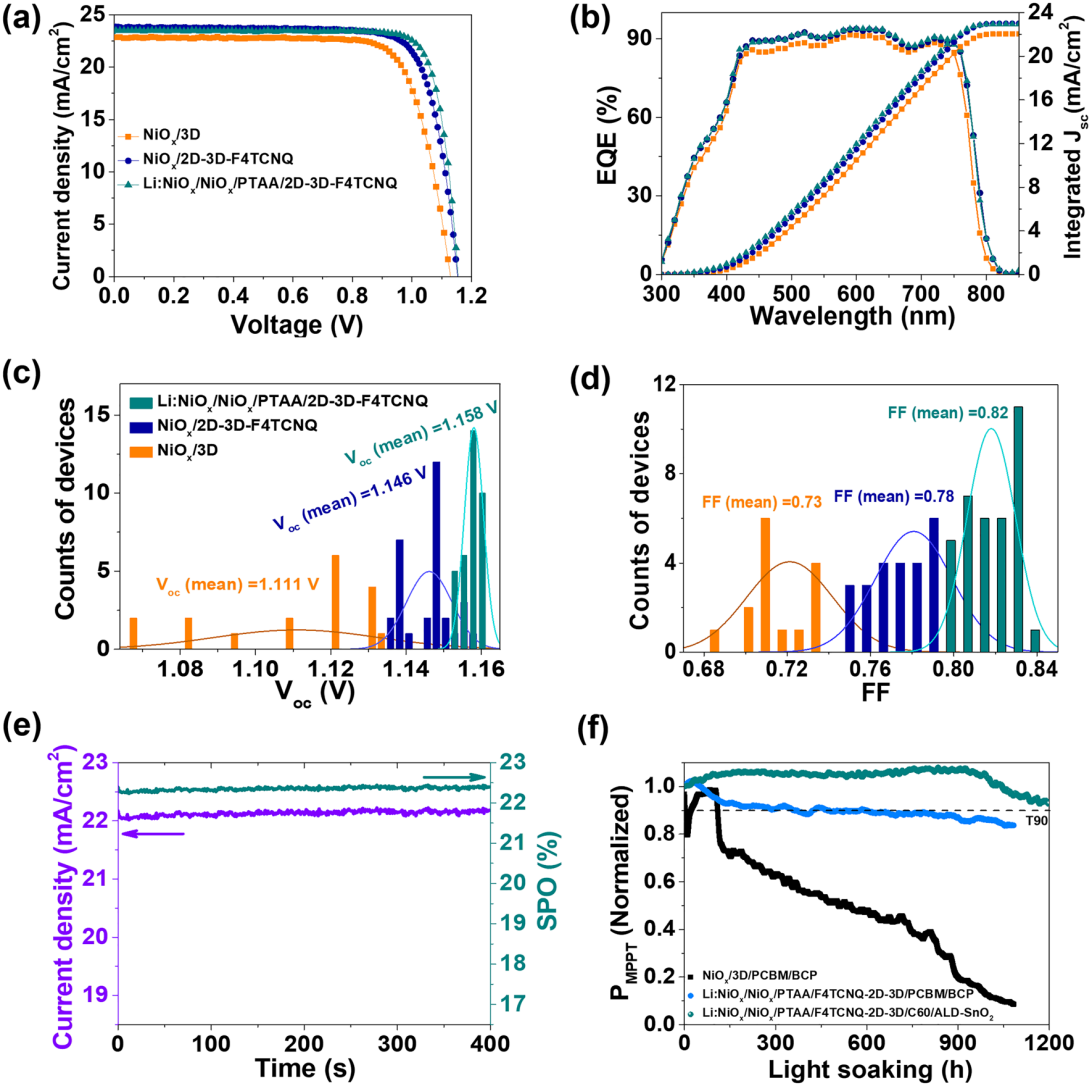
**a–b** *J-V* curves and corresponding EQE, **c–d** distribution of *V*_oc_ and FF for three types solar cells based on NiO_*x*_/3D perovskites (orange), NiO_*x*_/F4TCNQ-2D-3D perovskite composites (royal) and Li:NiO_*x*_/NiO_*x*_/F4TCNQ-2D-3D perovskite composites (dark cyan), **e–f** stabilized *J*_sc_ and SPO of bulk and interface modified cell, and stability test of power out at maximum power point under continuous one-Sun illumination in ambient-air conditions for three types of encapsulated devices based on different configurations: NiO_*x*_/3D perovskite/PCBM/BCP, Li:NiO_*x*_/NiO_*x*_/PTAA/F4TCNQ-2D-3D/PCBM/BCP and Li:NiO_*x*_/NiO_*x*_/PTAA/F4TCNQ-2D-3D/C60/ALD-SnO_2_

To evaluate the performance of NiO_*x*_-based solar cells, three types of device configuration were fabricated: FTO/NiO_*x*_/PTAA/mp-Al_2_O_3_/3D/2D/PCBM/BCP/Ag (control cell), FTO/NiO_*x*_/PTAA/mp-Al_2_O_3_/F4TCNQ-2D-3D/2D/PCBM/BCP/Ag (abbreviated for convenience as bulk modified cells) and FTO/Li:NiO_*x*_/NiO_*x*_/PTAA/mp-Al_2_O_3_/F4TCNQ-2D-3D/2D/PCBM/BCP/Ag (abbreviated for convenience as bulk and interface modified cell). As mentioned in previous works [34, 40, 41, 58, 59], it is important to avoid direct contact between NiO_*x*_ and perovskite for reducing interface defects-induced non-radiative recombination. Thus, after careful optimization of our control cells, an efficiency over 20% was obtained (Fig. 4a and Table 1). To build 2D-3D heterostructures, the OAmI content of 0.1 wt% was used in this work [38]. Compared to the control cell, the F4TCNQ-2D-3D composite-based cells (i.e., bulk modified cells) showed improved photovoltaic parameters (Figs. 4a and S9, Table S1). Surprisingly, the bulk and interface modified cells showed the best performance with a PCE, *V*_oc_ and FF of 23.06%, 1.162 V and 84% by optimizing the contents of Li:NiO_*x*_ (Figs. 4a and S10, Tables 1 and S2), which are the best performance for 1.56 electron volt bandgap perovskites to the best of our knowledge (Table S3). The integrated *J*_sc_ (23.01 mA cm^−2^) from EQE is almost agreement with measured *J*_sc_ (23.62 mA cm^−2^) from solar simulator (Fig. 4b). Compared to control and bulk modified cells, the bulk and interface modified cell cells showed highly reproducible performance with an average *V*_oc_ and FF of 1.158 V and 0.82, respectively (Fig. 4c-d). Figure 4e further shows highly stable *J*_sc_ (purple) and stabilized-power-output (SPO) (dark cyan) measured at the maximum power point voltage of 1.01 V for 400 s. From the space-charge-limited-current (SCLC) measurements, it can be observed that the 2D-3D-F4TCNQ perovskite films-based electron-only and hole-only devices have the lowest values of trap-filled limit voltage (${V}_{TFL}$) than that of 3D and 3D-F4TCNQ perovskite films-based devices (Fig. S11). Such results indicate interface defects-induced non-radiative recombination and energy-level barriers in devices can be remarkably suppressed by doping and charge-transport management strategies.

**Table 1:** Photovoltaic parameters of perovskite solar cells based on different types of device configurations

Device configuration	*V*_oc_ (V)	*J*_sc_ (mA cm^−2^)	FF	PCE (%)
FTO/NiO_*x*_/PTAA/mp-Al_2_O_3_/3D/2D/PCBM/BCP/Ag	1.134	22.87	0.78	20.23
FTO/NiO_*x*_/PTAA/mp-Al_2_O_3_/F4TCNQ-2D-3D/2D/PCBM/BCP/Ag	1.155	23.73	0.80	21.93
FTO/Li:NiO_*x*_/NiO_*x*_/PTAA/mp-Al_2_O_3_/F4TCNQ-2D-3D/2D/PCBM/BCP/Ag	1.162	23.62	0.84	23.06

We further examined the long-term operational stability of both 3D and F4TCNQ-2D-3D composite-based cells with encapsulation under continuous one-Sun illumination in ambient-air conditions. It can be observed that the 3D perovskite-based control devices with PCBM/BCP ETL showed large degradation almost 100% of the initial power after 1,000 h, but F4TCNQ-2D-3D perovskite composite-based cells with PCBM/BCP ETL were highly stable with T_90_ almost 1,000 h. More importantly, the F4TCNQ-2D-3D perovskite composite-based cells with C60/ALD-SnO_2_ ETL (C60 and SnO_2_ were prepared by evaporation and atomic layer deposition methods, respectively) exhibited excellent operational stability with T_90_ > 1,200 h. To confirm the contribution of chemical interactions and effective contact interfaces on the device stability improvement, XRD and XPS spectra were further measured for FTO/NiO_*x*_/3D perovskite (orange), FTO/Li:NiO_*x*_/NiO_*x*_/PTAA/3D perovskite (violet) and FTO/Li:NiO_*x*_/NiO_*x*_/PTAA/2D-3D-F4TCNQ (dark cyan) films treated at 85 °C under nitrogen atmosphere (Figs. S12-S13). Note that an obvious PbI_2_ peak was observed in above three films treated at 85 °C for 96 h, though there is no any PbI_2_ peak for all perovskite fresh films (Fig. S12a-b). However, the intensity of PbI_2_ peak in the FTO/NiO_*x*_/3D perovskite film extremely increases compared with other two films. We also found that iodide (or iodine) concentration (I 3*d* peak) in the FTO/NiO_*x*_/3D perovskite film dramatically increases with thermal aging time than other two films (Fig. S13a-c), which indicates the iodide (or iodine) ions heavily migrated towards the surface of perovskite film. Surprisingly, the intensity of I 3*d* peaks in FTO/Li:NiO_*x*_/NiO_*x*_/PTAA/2D-3D-F4TCNQ film are almost same in both conditions (Fig. S13c). Besides, no obvious Pb^0^ peaks could be observed in FTO/Li:NiO_*x*_/NiO_*x*_/PTAA/2D-3D-F4TCNQ film (Fig. S13f), but other two perovskite films show two stronger metallic Pb^0^ peaks as shown in Fig. S13d-e. Such results indicate that innovation design of device interfaces and passivation of perovskite defects are significant to further enhance device stability.

## Conclusions

In summary, we have fabricated NiO_*x*_-based graded inverted perovskite solar cells by hole-transport management strategy. The defective contact interfaces presented at between hole-selective contacts (HSCs) and the perovskite-active layer (PAL) are remarkably suppressed by efficient interface contact, cascaded energy-level structures, and p-type molecule-doped composites with 2D-3D perovskites with strong chemical interaction and bonding, resulting in fast hole extraction and transport, and efficient interface defect passivation. The optimized cell obtained an efficiency over 23% with a high FF of 84% and *V*_oc_ of 1.162 V, and also showed excellent operational stability with T_90_ over 1,200 h. We believe that such a hole-transport management strategy is an efficient route to suppress heterointerface defects-induced non-radiative recombination for achieving close to theoretical *V*_oc_ and FF in NiO_*x*_ based inverted PSCs.

### Supplementary Information

Below is the link to the electronic supplementary material.

Supplementary file1 (PDF 943 kb)
