# Supplementary material for: Hole-Transport Management Enables 23%-Efficient and Stable Inverted Perovskite Solar Cells with 84% Fill Factor
Source: Nanomicro Lett. 2023 Apr 30;15:117. doi: 10.1007/s40820-023-01088-4 (PMC10149558; doi:10.1007/s40820-023-01088-4)
Supplement: Supplementary file 1 — Supplementary file1 (PDF 943 kb) [file 40820_2023_1088_MOESM1_ESM.pdf]

Supporting Information for

Hole-Transport Management Enables 23%-Efficient and Stable Inverted Perovskite Solar Cells with 84% Fill Factor

Liming Liu [1, 3, #], Yajie Ma [1, 3, #], Yousheng Wang[1, 2, 3, *], Qiaoyan Ma[1, 3], Zixuan Wang[1, 3], Zigan Yang[1, 3], Meixiu Wan[1, 3], Tahmineh Mahmoudi[4], Yoon-Bong Hahn[4], Yaohua Mai[1, 2, 3, *]

[1]Institute of New Energy Technology, College of Information Science and Technology, Jinan University, Guangzhou 510632, P. R. China

[2]Guangdong Mellow Energy Co., Ltd., Guangzhou 510630, P. R. China

[3]Key Laboratory of New Semiconductors and Devices of Guangdong Higher Education Institutes, Jinan University, Guangzhou 510632, P. R. China

[4]School of Semiconductor and Chemical Engineering, Solar Energy Research Center, Jeonbuk National University, 567 Baekjedaero, Deokjin-gu, Jeonju-si, Jeollabuk-do, 54896 Republic of Korea

# Liming Liu and Yajie Ma contributed equally to this work.

*Corresponding authors. E-mail: wangys0120@jnu.edu.cn (Y. Wang); yaohuamai@jnu.edu.cn (Y. Mai)

Supplementary Figures and Tables

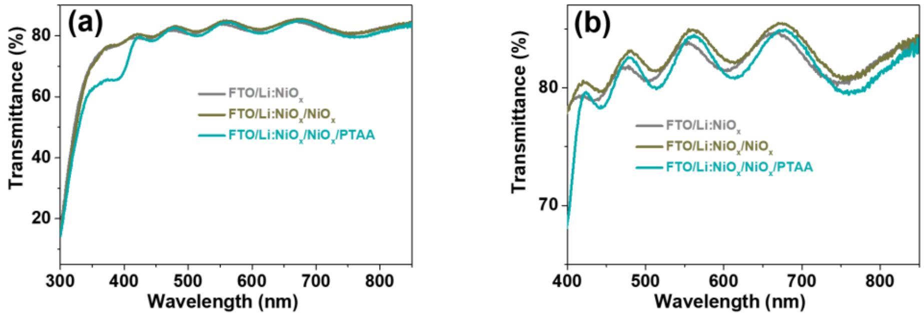

Fig. S1 UV transmittance for three hole-contact films: FTO/Li:NiO$_x$, FTO/Li:NiO$_x$/NiO$_x$ and FTO/Li:NiO$_x$/NiO$_x$

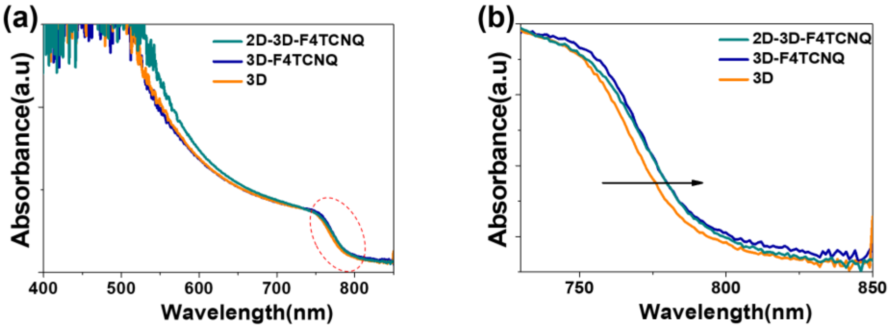

Fig. S2 UV-vis spectra for three perovskite films: 3D, F4TCNQ-3D and F4TCNQ-2D-3D composites

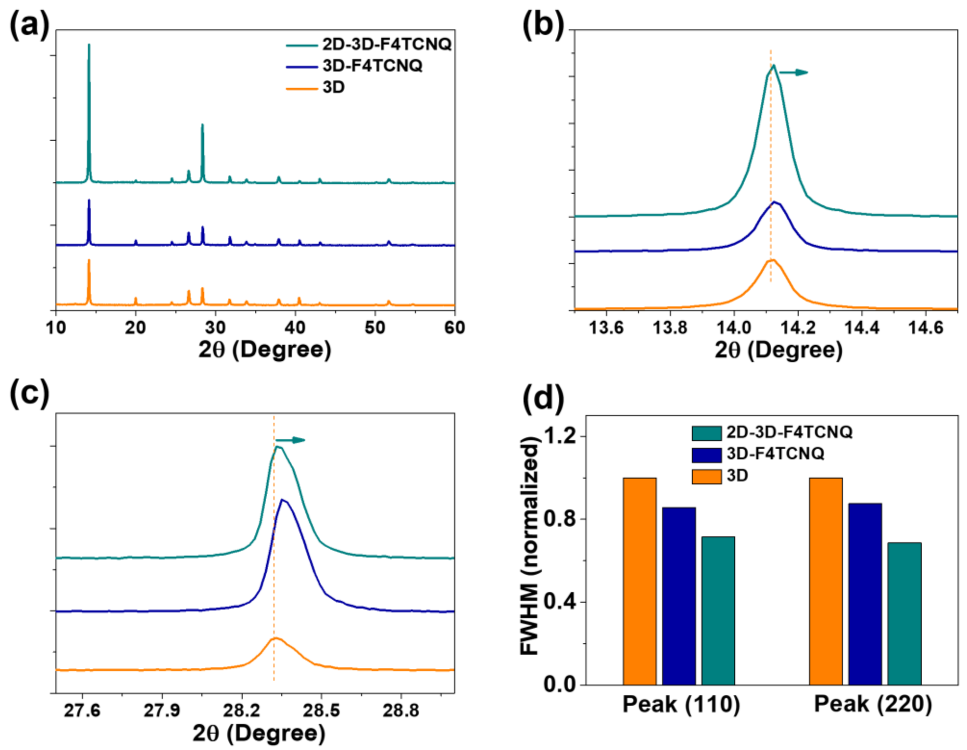

Fig. S3 (**a**) X-ray diffraction patterns, (**b**) peak (110), (**c**) peak (220) and (**d**) corresponding FWHM values for three perovskite films: 3D, F4TCNQ-3D and F4TCNQ-2D-3D composites

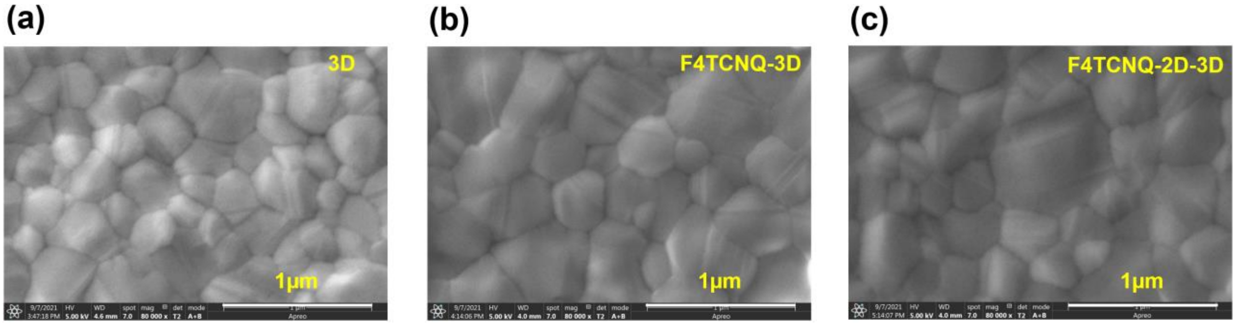

Fig. S4 SEM images for surface morphology of three perovskite films: (**a**) 3D, (**b**) F4TCNQ-3D and (**c**) F4TCNQ-2D-3D composites, respectively

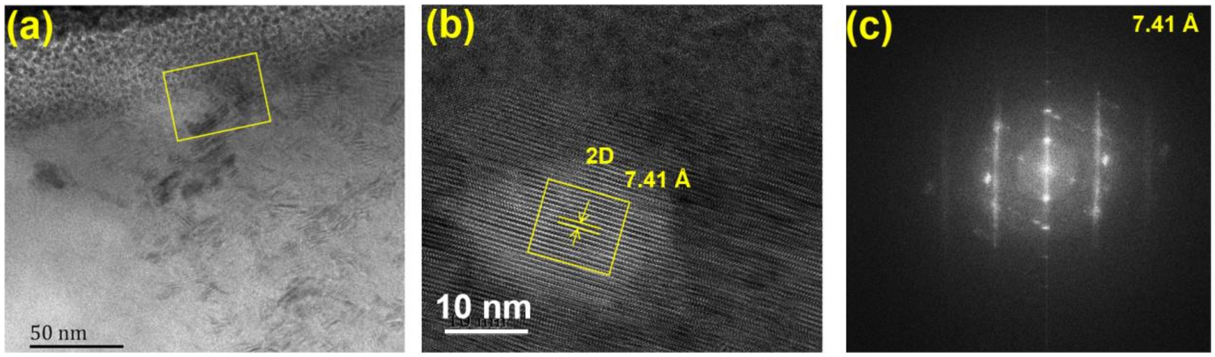

Fig. S5 The FIB-cutted cross-sectional images for (**a**) 2D/3D heterointerface, (**b**) selected region of HR-TEM and (**c**) corresponding inverse FFT images for 2D crystals, respectively

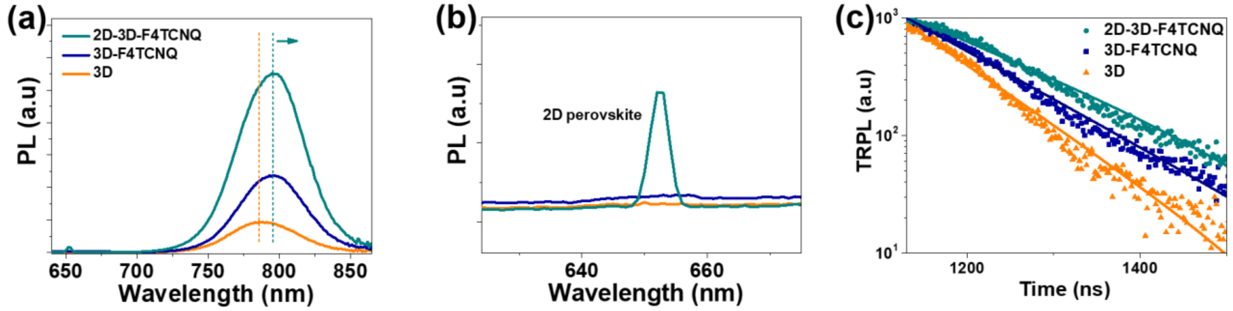

Fig. S6 (**a**) PL spectra, (**b**) 2D peak and (**c**) TRPL spectra for three perovskite films: 3D (orange), F4TCNQ-3D (royal) and F4TCNQ-2D-3D composites (dark cyan), respectively

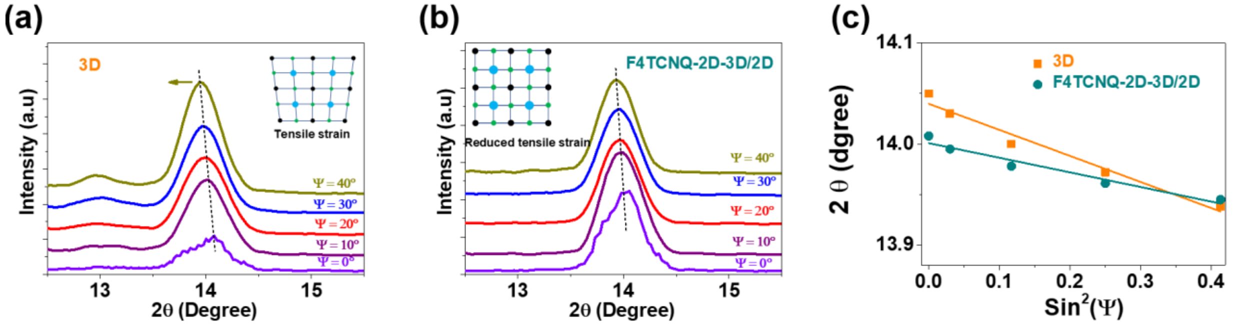

Fig. S7 (**a-b**) GIXRD spectra at different tilt angles for 2θ at 14.07°, (**c**) linear fit of residual strain as a function of $\sin^2\psi$ for pristine 3D perovskite and F4TCNQ-2D-3D composite films, respectively

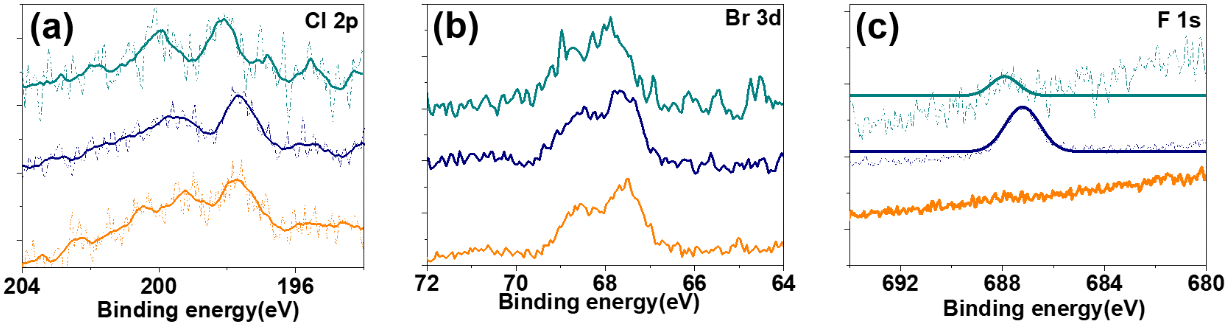

Fig. S8 XPS spectra of (**a**) Cl 2p, (**b**) Br 3d and (**c**) F 1s for 3D (orange), F4TCNQ-3D (royal) and F4TCNQ-2D-3D composites (dark cyan), respectively

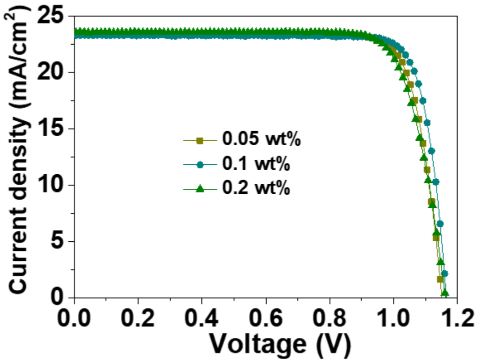

Fig. S9 J-V curves of solar cells based on various contents of F4TCNQ

Table S1 Photovoltaic parameters of perovskite solar cells for different contents of F4TCNQ

Contents of F4TCNQ	V_{oc} (V)	J_{sc} (mA/cm^2)	FF	PCE (%)
0.05 wt%	1.134	22.70	0.81	20.85
0.1 wt%	1.159	23.20	0.84	22.48
0.2 wt%	1.160	23.47	0.79	21.50

Table S2 Photovoltaic parameters of perovskite solar cells based on different contents of Li ratios

Ratio of V_{Li}/V_{Ni} precursor	V_{oc} (V)	J_{sc} (mA/cm^2)	FF	PCE (%)
1:2	1.135	22.70	0.81	20.85
1:3	1.148	23.42	0.81	21.77
1:4	1.162	23.60	0.82	22.49
1:5	1.160	23.47	0.79	21.50

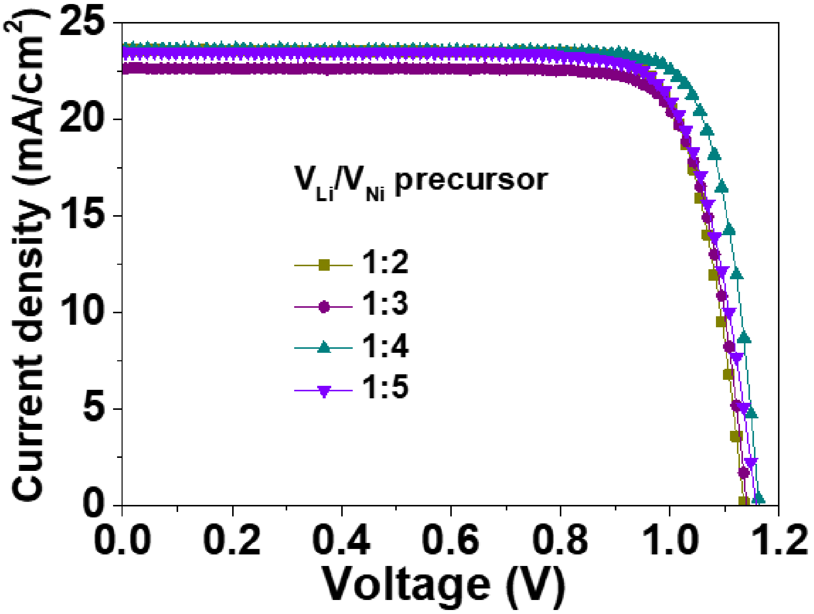

Fig. S10 J-V curves of solar cells based on various contents of lithium in $Li:NiO_x$ precursors

Table S3 Summary of photovoltaic parameters of NiO_x-based inverted perovskites solar cells with PCEs over 22% based on different band gaps of perovskites

Perovskite	E_g (eV)	PCE (%)	V_{oc} (V)	J_{sc} (mA/cm^2)	FF	Ref.
$FA_{0.93}MA_{0.07}Pb(I_{0.92}Br_{0.08})_{3-x}Cl_x$	1.56	23.06	1.162	23.62	0.84	This work
$Cs_{0.05}FA_{0.85}MA_{0.1}PbI_3$	1.55	22.90	1.170	23.42	0.82	[S1]
$(FAPbI_3)_{0.92}(MAPbBr_3)_{0.08}$	1.57	24.09	1.204	23.70	0.84	[S2]
$Cs_{0.05}(MA_{0.15}FA_{0.85})_{0.95}Pb(I_{0.85}Br_{0.15})_3$	1.59	22.62	1.130	23.75	0.84	[S3]
$Cs_{0.05}FA_{0.83}MA_{0.12}PbI_{2.62}Br_{0.38}$	1.60	22.21	1.161	23.50	0.82	[S4]
$Cs_{0.05}FA_{0.85}MA_{0.1}PbI_3$	1.55	23.91	1.151	24.90	0.83	[S5]
$Cs_{0.03}(FA_{0.85}MA_{0.15})_{0.97}Pb(I_{0.85}Br_{0.15})_3$	1.60	22.00	1.160	23.31	0.81	[S6]
$Cs_{0.05}(FA_{0.85}MA_{0.15})_{0.95}Pb(I_{0.85}Br_{0.15})_3$	1.59	22.11	1.131	23.24	0.84	[S7]
$MAPbI_3$ nanocapsules	1.63	22.10	1.150	23.25	0.82	[S8]
$Cs_{0.05}(FA_{0.9}MA_{0.1})_{0.95}Pb(I_{0.9}Br_{0.1})_3$	1.57	22.10	1.130	24.30	0.81	[S9]
$FA_{0.95}Cs_{0.05}PbI_3$	1.53	23.49	1.138	25.38	0.84	[S10]
$FA_{0.92}Cs_{0.08}PbI_3$	1.54	23.40	1.160	24.80	0.81	[S11]

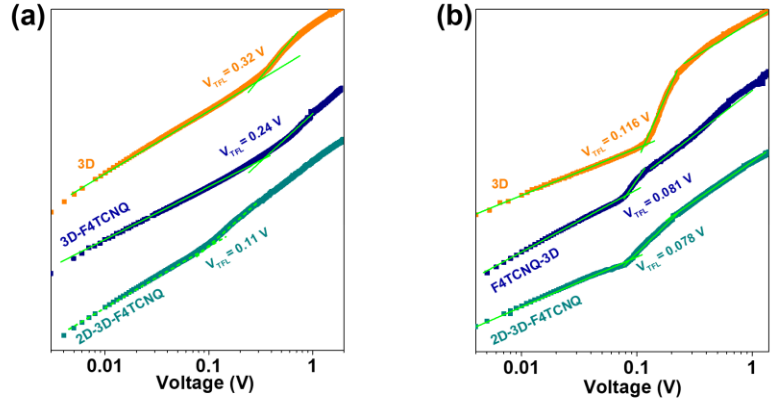

Fig. S11 I-V curves of (**a**) electron-only devices and (**b**) hole-only devices based on pristine 3D (orange), F4TCNQ composite-3D (royal) and F4TCNQ-2D-3D composites (dark cyan) composite films

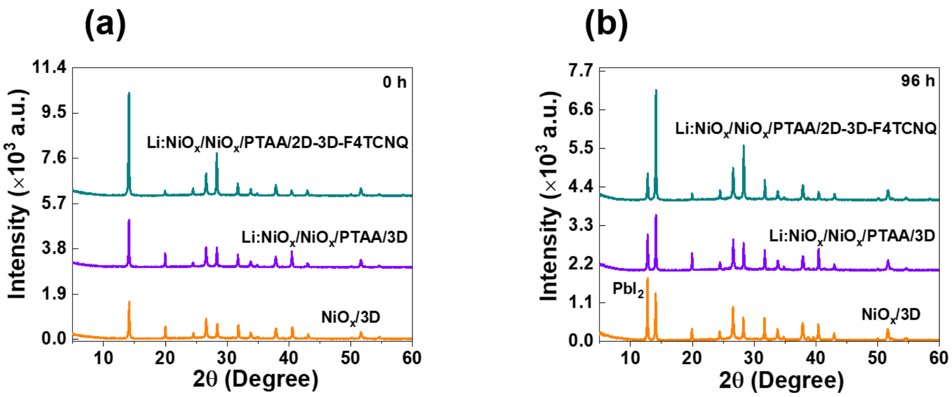

Fig. S12 XRD of NiO_x/3D-perovskite (orange), Li:NiO_x/NiO_x/PTAA/3D-perovskite (violet) and Li:NiO_x/NiO_x/PTAA/2D-3D-F4TCNQ perovskite (dark cyan) films treated at 85℃ temperature for different time (**a**) 0 h and (**b**) 96 h, respectively

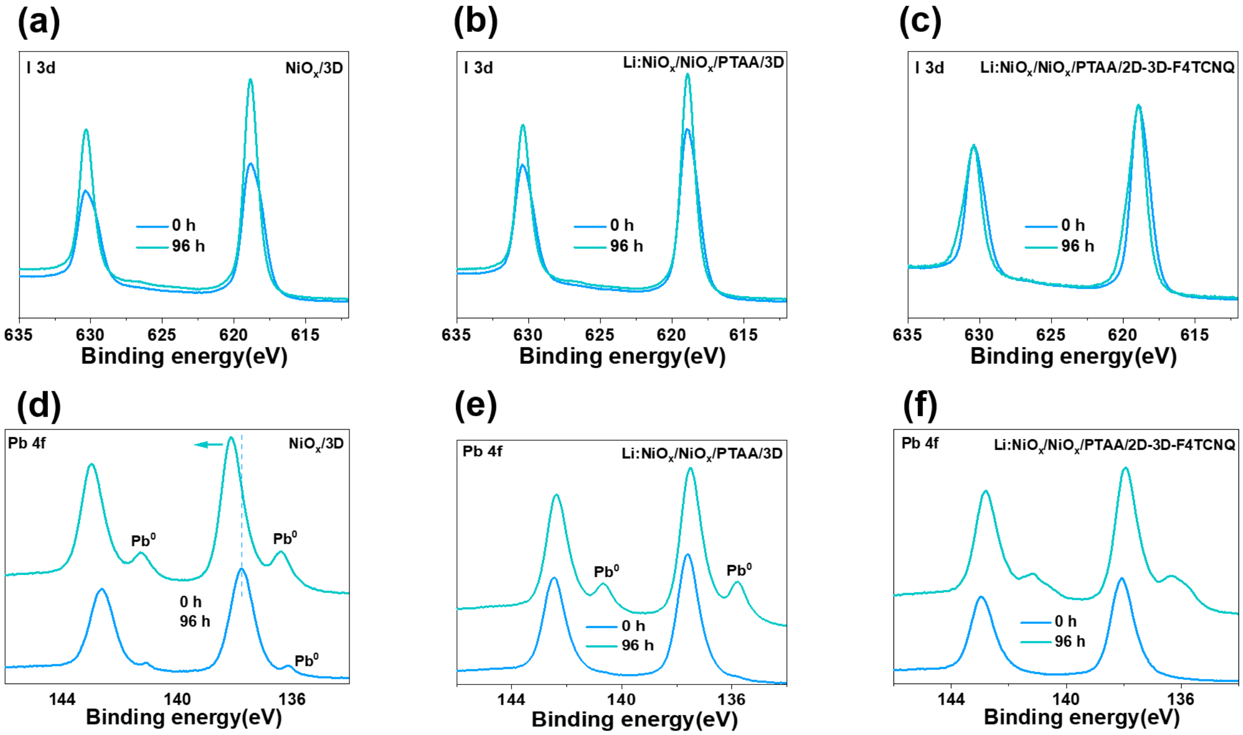

Fig. S13 XPS spectra of I 3d (**a-c**) and Pb 4f (**d-f**) for NiO_x/3D-perovskite, Li:NiO_x/NiO_x/PTAA/3D-perovskite and Li:NiO_x/NiO_x/PTAA/2D-3D-F4TCNQ perovskite films treated at 85℃ temperature for different time, respectively

Supplementary References

[S1] B. Chen, H. Chen, Y. Hou, J. Xu, S. Teale et al., Passivation of the buried interface via preferential crystallization of 2D perovskite on metal oxide transport layers. Adv. Mater. **33**, 2103394 (2021). https://doi.org/10.1002/adma.202103394

[S2] Y. Zhang, C. Li, E. Bi, T. Wang, P. Zhang et al., Efficient inverted perovskite solar cells with a low-dimensional halide/perovskite heterostructure. Adv. Energy Mater. **12**, 2202191 (2022). https://doi.org/10.1002/aenm.202202191

[S3] S. Wang, Y. Li, J. Yang, T. Wang, B. Yang et al., Critical role of removing impurities in nickel oxide on high-efficiency and long-term stability of inverted perovskite solar cells. Angew. Chem. Int. Ed. **61**, e2021165 (2022). https://doi.org/10.1002/anie.202116534

[S4] Z. Wang, L. Liu, Y. Wang, Y. Ma, Z. Yang et al., Green antisolvent-mediators stabilize perovskites for efficient NiO_x-based inverted solar cells with Voc approaching 1.2V. Chem. Eng. J. **457**, 141204 (2023). https://doi.org/10.1016/j.cej.2022.141204

[S5] H. Chen, S. Teale, B. Chen, Y. Hou, L. Grater et al., Quantum-size-tuned heterostructures enable efficient and stable inverted perovskite solar cells. Nat. Photon. **16,** 352-358 (2022). https://doi.org/10.1038/s41566-022-00985-1

[S6] W. Chen, B. Han, Q. Hu, M. Gu, Y. Zhu et al., Interfacial stabilization for inverted perovskite solar cells with long-term stability. Sci. Bull. **66**, 991-1002 (2021). https://doi.org/10.1016/j.scib.2021.02.029

[S7] Q. Cao, Y. Li, H. Zhang, J. Yang, J. Han et al., Efficient and stable inverted perovskite solar cells with very high fill factors via incorporation of star-shaped polymer. Sci. Adv. **7**, eabg0633 (2021). https://doi.org/10.1126/sciadv.abg0633

[S8] Z. Huang, X. Hu, Z. Zhao, X. Meng, M. Su et al., Releasing nanocapsules for high-throughput printing of stable perovskite solar cells. Adv. Energy Mater. **11**, 2101291 (2021) https://doi.org/10.1002/aenm.202101291

[S9] B. Niu, H. Wu, J. Yin, B. Wang, G. Wu et al., Mitigating the lead leakage of high-performance perovskite solar cells via in situ polymerized networks. ACS Energy Lett. **6**, 3443-3449 (2021). https://doi.org/10.1021/acsenergylett.1c01487

[S10] M. Li, H. Li, Q. Zhuang, D. He, B. Liu et al., Stabilizing perovskite precursor by synergy of functional groups for NiO_x-based inverted solar cells with 23.5 % efficiency. Angew. Chem. Int. Ed. **61**, e202206914 (2022). https://doi.org/10.1002/anie.202206914

[S11] M. Du, S. Zhao, L. Duan, Y. Cao, H. Wang et al., Surface redox engineering of vacuum-deposited NiO_x for top-performance perovskite solar cells and modules. Joule **6**, 1931-1943 (2022). https://doi.org/10.1016/j.joule.2022.06.026
